# Intraoperative Frontal Alpha-Band Power Correlates with Preoperative Neurocognitive Function in Older Adults

**DOI:** 10.3389/fnsys.2017.00024

**Published:** 2017-05-08

**Authors:** Charles M. Giattino, Jacob E. Gardner, Faris M. Sbahi, Kenneth C. Roberts, Mary Cooter, Eugene Moretti, Jeffrey N. Browndyke, Joseph P. Mathew, Marty G. Woldorff, Miles Berger, the MADCO-PC Investigators, Miles Berger

**Affiliations:** ^1^Center for Cognitive Neuroscience, Duke University Durham, NC, USA; ^2^Department of Psychology and Neuroscience, Duke University Durham, NC, USA; ^3^Department of Anesthesiology, Duke University Medical Center Durham, NC, USA; ^4^Department of Psychiatry and Behavioral Sciences, Duke University Medical Center Durham, NC, USA; ^5^Department of Neurobiology, Duke University Medical Center Durham, NC, USA

**Keywords:** cognitive function, aging, alpha oscillations, anteriorization, EEG, general anesthesia, isoflurane, propofol

## Abstract

Each year over 16 million older Americans undergo general anesthesia for surgery, and up to 40% develop postoperative delirium and/or cognitive dysfunction (POCD). Delirium and POCD are each associated with decreased quality of life, early retirement, increased 1-year mortality, and long-term cognitive decline. Multiple investigators have thus suggested that anesthesia and surgery place severe stress on the aging brain, and that patients with less ability to withstand this stress will be at increased risk for developing postoperative delirium and POCD. Delirium and POCD risk are increased in patients with lower preoperative cognitive function, yet preoperative cognitive function is not routinely assessed, and no intraoperative physiological predictors have been found that correlate with lower preoperative cognitive function. Since general anesthesia causes alpha-band (8–12 Hz) electroencephalogram (EEG) power to decrease occipitally and increase frontally (known as “anteriorization”), and anesthetic-induced frontal alpha power is reduced in older adults, we hypothesized that lower intraoperative frontal alpha power might correlate with lower preoperative cognitive function. Here, we provide evidence that such a correlation exists, suggesting that lower intraoperative frontal alpha power could be used as a physiological marker to identify older adults with lower preoperative cognitive function. Lower intraoperative frontal alpha power could thus be used to target these at-risk patients for possible therapeutic interventions to help prevent postoperative delirium and POCD, or for increased postoperative monitoring and follow-up. More generally, these results suggest that understanding interindividual differences in how the brain responds to anesthetic drugs can be used as a probe of neurocognitive function (and dysfunction), and might be a useful measure of neurocognitive function in older adults.

## Introduction

Over 16 million Americans over age 60 undergo general anesthesia for surgery each year, after which up to 40% likely develop postoperative delirium and/or cognitive dysfunction (POCD; Inouye et al., 2014; Berger et al., 2015). Although POCD and delirium are distinct syndromes assessed by different instruments, each is associated with decreased quality of life (Phillips-Bute et al., 2006; Basinski et al., 2010; Naidech et al., 2013), increased 1-year mortality, long-term cognitive decline, and possible increased risk of developing dementia (Inouye et al., 2014, 2016; Berger et al., 2015). Further, poor preoperative cognitive function is a risk factor for developing POCD (Bekker et al., 2010; Silbert et al., 2015; reviewed in Berger et al., 2015) and may be a risk factor for postoperative delirium (Greene et al., 2009; but also see Scott et al., 2015). Thus, identifying patients with poor preoperative cognitive function could be used to risk-stratify patients at high risk of POCD and delirium, and to direct resources toward prevention, treatment, and management efforts for these high-risk patients.

Formally measuring preoperative cognitive function, however, would require detailed cognitive testing, which is not part of the 2012 American Society of Anesthesiologists practice advisory for preanesthesia evaluation (Committee on Standards and Practice Parameters et al., 2012). Performing this additional cognitive testing on >16 million older Americans would be logistically complex and financially costly given the financial pressures on the American health care system. For example, even if each preoperative cognitive assessment only cost $100, the total cost of performing these assessments on 16 million patients would be $1.6 billion.

To deal with this issue, researchers have employed brief, handwritten cognitive assessments (Culley et al., 2016), as well as computerized cognitive assessment tools. However, even brief, handwritten cognitive assessments take time to interpret (and would thus be costly to apply on a systematic basis), and computerized cognitive assessments may be difficult to use for older patients with poor computer literacy.

Another potential approach to assessing preoperative neurocognitive status could be to measure how a patient’s brain responds to intraoperative anesthetic administration. We and others have proposed that anesthesia and surgery place severe stress on the aging brain, and that an individual’s ability to withstand this stress can serve as an assay for their cognitive resilience (Nadelson et al., 2014; Berger et al., 2015), which implies that we may be able to measure signs of decreased neurocognitive function by carefully assessing how the brain responds to anesthesia. Indeed, the human brain’s response to general anesthesia can be measured noninvasively with scalp electroencephalogram (EEG), and there are several commercially available intraoperative EEG-based brain function monitors. Further, since patients with Alzheimer’s Disease (AD) and Mild Cognitive Impairment (MCI) have altered EEG patterns even while awake (Prichep et al., 1994; Kwak, 2006; van der Hiele et al., 2007), we hypothesized that the stress of anesthesia and surgery might give rise to altered EEG patterns in patients with poor preoperative cognitive function. More generally, we hypothesized that certain features of the EEG measured intraoperatively might correlate with, or be predictive of, a wide range of cognitive functional statuses.

The EEG can be divided into frequency bands, of which one of the most prominent is alpha (8–12 Hz; Berger, 1929). In the awake state—particularly with the eyes closed—this alpha activity is maximal over parieto-occipital scalp locations, which is thought to reflect rhythmic, reciprocal interactions between the thalamus (particularly the lateral geniculate and pulvinar nuclei) and visual areas in the occipital and parietal cortices (Lopes da Silva et al., 1980; Steriade et al., 1990; Hughes et al., 2011). Functionally, awake alpha has been associated with levels of arousal, relative cortical deactivation (Pfurtscheller et al., 1996) or inhibition (Jensen and Mazaheri, 2010; Foxe and Snyder, 2011), and attention (Grent-’t-Jong et al., 2011; Liu et al., 2016), and is thus an important factor in cognitive function. Indeed, measures of awake alpha have been found to be decreased in patients with cognitive deficits such as AD and MCI (van der Hiele et al., 2007; reviewed in Rossini et al., 2007).

Alpha power displays a significant distributional shift under general anesthesia, decreasing occipitally and increasing frontally (a process termed “anteriorization”; Tinker et al., 1977; John et al., 2001; Purdon et al., 2013). The mechanisms of this alpha anteriorization are not well understood. Modeling work suggests that anteriorization might arise from the differential effect of anesthetic drugs on distinct thalamic nuclei (Ching et al., 2010; Vijayan et al., 2013), which then alters interactions between the thalamus and cortex, although differential effects on distinct regions of cortex (e.g., occipital vs. frontal) could also be a factor. These mechanisms are thought to give rise to large-scale, anesthetic-induced changes to alpha topography, which correlate with anesthetic-induced unconsciousness (Tinker et al., 1977; although also see Blain-Moraes et al., 2016, which suggested that anteriorization occurs at higher doses of anesthetics than are necessary for loss of consciousness). Since thalamocortical interactions are critical for consciousness and cognitive function (Castaigne et al., 1981; Blumenfeld, 2010; Berger and García, 2016), and lower awake alpha power has been shown to be associated with cognitive deficits (reviewed in Rossini et al., 2007), we hypothesized that the ability of anesthesia to induce strong frontal alpha oscillations might also be a predictor or correlate of a patient’s preoperative cognitive function.

In addition, it is known that alpha power in the awake state decreases with age (Roubicek, 1977; reviewed in Rossini et al., 2007), and Purdon et al. (2015a) recently showed that anesthetic-induced frontal alpha power also declines as a function of age. These decreases in alpha power might be due to cortical atrophy or other factors related to typical brain aging (Purdon et al., 2015a), such as changes in the cholinergic basal forebrain system (Sarter and Bruno, 1998) that is thought to play an important role in alpha generation (Lorincz et al., 2009), conditions that would likely also impact on the functional capacity of the affected brain regions. Purdon et al. (2015a) also observed wide variation across older patients in frontal alpha power under general anesthesia, which suggests the possibility that such variation among anesthetized older adults might correlate with preoperative neurocognitive function.

Notably, however, the work by Purdon et al. (2015a) used only four channels of prefrontal EEG from the SEDLine monitor (Masimo Corporation, Irvine, CA, USA), and thus could not fully capture scalp topography changes in alpha power under general anesthesia. Moreover, previous studies had not examined the relationship between intraoperative alpha power (frontally or at other locations) and preoperative cognitive function. Accordingly, our main objective here was to examine the hypothesis that intraoperative frontal alpha power under general anesthesia would correlate with preoperative neurocognitive function. As a secondary objective, we also examined whether either frontal EEG power in other frequency bands or alpha power at other scalp locations would correlate with preoperative neurocognitive function. To pursue these objectives, we took advantage of an ongoing prospective cohort study (NCT01993836) that included preoperative cognitive testing by having a subset of these study patients also undergo intraoperative EEG recordings.

## Materials and Methods

### Study Population

The present study protocol was approved by the Duke University Medical Center Institutional Review Board as part of a parent cohort study that was registered with clinicaltrials.gov (NCT01993836). All subjects gave written informed consent in accordance with the Declaration of Helsinki. For the parent study, we enrolled patients age 60 and over undergoing non-cardiac, non-neurologic surgery under general anesthesia that was scheduled for at least 2 h duration and with a planned overnight hospital stay. All patients received propofol for anesthetic induction, and either propofol or isoflurane as the primary anesthetic maintenance drug. We excluded patients who had a personal or family history of malignant hyperthermia or other medical contraindication to receive isoflurane or propofol. There were no exclusions for preoperative cognitive status or prior neurologic disease (such as MCI, dementia due to AD, or stroke). However, all enrolled patients needed to be able to, and were able to, complete our cognitive test battery (described below), which required intact language function.

In patients in the bispectral index (BIS) EEG recording group of this study, a BIS Quatro sensor (Medtronic/Covidien, Dublin, Ireland) was placed on the left side of the forehead to record raw EEG data. A second BIS Quatro sensor was placed on the right side of the forehead, connected to the anesthesia display system in the operating room, and used to titrate anesthetic depth to maintain a BIS index range of 40–60, in combination with other intraoperative hemodynamic and physiologic measurements, as per standard clinical practice at Duke. In patients in the 32-channel EEG recording group, a BIS Quatro sensor was placed in the nasal montage position as previously described (Nelson et al., 2013)—rather than on the forehead as in the BIS EEG group—and used to titrate anesthetic depth as described above. The anesthesiologists, anesthesiology residents, and nurse anesthetists caring for these patients were blinded to the patients’ preoperative cognitive testing data, and to their intraoperative 32-channel or left frontal BIS EEG data and recordings. The baseline characteristics for the 32-channel and BIS EEG groups are described in Tables 1, 2, respectively.

**Table 1:** **Baseline characteristics of patients who underwent intraoperative 32-channel electroencephalogram (EEG) recordings**.

Age	69 (5.5)
Sex	8 Male, 7 Female
Surgery type	General abdominal—5; Orthopedic—2; Urologic—3; Gynecologic—1; Thoracic—4
Surgery case length (min)	285 (110)
ASA status	ASA 1–0; ASA 2–0; ASA 3–15; ASA 4–0
Maintenance anesthetic type	Propofol (TIVA)—9; Isoflurane—6
Age-adjusted end tidal isoflurane MAC fraction*	0.95 (0.27)
Total propofol dose (mg)*	2046 (1075)
Average BIS index value	49 (8)
Fentanyl dose (mcg)	190 (102)
# of patients who received dexmedetomidine	2
# of patients who received ketamine	2

Continuous data are presented as means (SD); categorical data are presented in list format. ASA, American Society of Anesthesiology; TIVA, total intravenous anesthesia. *Values listed are the mean doses in those patient who received the indicated anesthetic drug (i.e., isoflurane or propofol) for anesthetic maintenance during the case.

**Table 2:** **Baseline characteristics of patients who underwent bispectral index (BIS) EEG recordings**.

Age	69 (6)
Sex	24 Male, 11 Female
Surgery type	General abdominal—7; Orthopedic—7; Urologic—13; Gynecologic—1; Plastic—3; Thoracic—4
Surgery case length (mins)	198 (98)
ASA status	ASA 1–1; ASA 2–8; ASA 3–25; ASA 4–1
Maintenance anesthetic type	Propofol (TIVA)—16; Isoflurane—19
Age-adjusted end tidal isoflurane MAC fraction*	0.79 (0.12)
Total propofol dose (mg)*	1339 (799)
Average BIS index value	50 (6)
Fentanyl dose (mcg)	208 (89)
# of patients who received dexmedetomidine	5
# of patients who received ketamine	8

Continuous data are presented as means (SD); categorical data are presented in list format. ASA, American Society of Anesthesiology; TIVA, total intravenous anesthesia. *Values listed are the mean doses in those patient who received the indicated anesthetic drug (i.e., isoflurane or propofol) for anesthetic maintenance during the case.

The purpose of this sub-study was to evaluate the possible correlation between intraoperative frontal alpha power and preoperative cognitive function. Since we were unaware of any previous studies that had evaluated this question, there was no solid preliminary data to guide an *a priori* sample size calculation for this sub-study. Accordingly, for this sub-study, we consented all patients enrolled in the parent study (NCT01993836) from 29 June 2015 to 7 November 2016 to undergo intraoperative EEG recordings, in addition to preoperative cognitive testing (see section below) that was part of the parent study.

### EEG Data Collection, Preprocessing, and Spectral Analysis

In 17 consecutively enrolled patients, we recorded EEG from 32 electrodes embedded in a 64-electrode cap custom designed for extended scalp coverage (Woldorff et al., 2002; BrainAmp MR Plus, Brain Products GmbH, Gilching, Germany). The EEG data from 2 of these patients had to be excluded from this analysis due to excessive artifacts (*n* = 1) or incomplete data collection (*n* = 1), leaving 15 patients with 32-channel EEG data for analysis. EEG was recorded during the preoperative period (3 min each with eyes open and with eyes closed) and during general anesthesia and surgery, which was the primary focus of our analysis based on our hypothesis that general anesthesia may serve as a stressor that brings out differences in preoperative cognition. In addition, few clinicians obtain preoperative EEG recordings before the induction of general anesthesia, and instead apply EEG monitors only after patients have been anesthetized. Accordingly, here we focused on the correlation between intraoperative EEG measures under general anesthesia and preoperative cognitive function.

All signals were recorded with a band-pass filter of 0.016–250 Hz (the 250 Hz low-pass filter was a fifth-order Butterworth with a fairly sharp 30 dB/octave roll-off), digitized at 500 Hz, and referenced to scalp-site Cz. Electrode impedances were lowered to below 20 kΩ before recording by light abrasion of the scalp locations with a moderately gritty electrode paste (Abralyte 2000, EASYCAP GmbH, Herrsching, Germany). After recording, the EEG data were band-pass filtered from 0.1 Hz to 60 Hz and downsampled to 250 Hz. We also re-referenced the data to the algebraic average of the left and right mastoid electrodes to facilitate comparison with previous research examining alpha topographies using a full-scalp electrode montage (e.g., Grent-’t-Jong et al., 2011; Rohenkohl and Nobre, 2011, and as recommended by Cohen, 2014; Luck, 2014). We selected EEG data beginning after surgical incision and either until the end of anesthetic maintenance (i.e., before emergence; *n* = 4) or until 2 h after the start of surgery (*n* = 11).

Raw EEG data from each patient was examined by an observer blinded to the patient’s cognitive status to identify cases with grossly significant EEG data artifacts; additional data preprocessing was then used to remove these artifacts as described below. Due to unusually high artifactual drift in 2 patients, their data were additionally high-pass filtered with a half-amplitude cutoff at 1 Hz and a 1 Hz transition zone (from 0.5 Hz to 1.5 Hz). As this filter attenuates signals in the lower delta band (between 1 Hz and 1.5 Hz) and could thus affect our results, we also re-ran our analyses by: (1) using the same 0.1 Hz high-pass filter for every patient; and (2) excluding these 2 patients from the analyses entirely. Both of these approaches yielded nearly identical results as are reported here. Due to excessive noise artifacts in the mastoid electrodes of 1 patient, we used nearest-neighbor interpolation to estimate the activity of those channels before re-referencing. We segmented the data into 3 s epochs, and epochs with voltage artifacts greater than ±100 μV were excluded from further analysis. Independent component analysis (ICA) was used to identify and remove cardiac, electrocautery, and EMG artifacts from the EEG data in 3 patients. Channels with noise artifacts occurring in significant portions of the recording session were either interpolated based on the surrounding channels using spherical spline interpolation (Perrin et al., 1989) or removed. We analyzed all artifact-free data following standard methods for multi-electrode montage recordings (Cohen, 2014; Luck, 2014). All EEG preprocessing was performed using the EEGLAB toolbox (Delorme and Makeig, 2004) and custom scripts in MATLAB (ver. 2013b, The MathWorks, Inc., Natick, MA, USA).

For 38 additional consecutively enrolled patients, frontal EEG was recorded using a BIS Quatro sensor placed on the left side of the forehead, which was connected to a BIS A-2000 monitor (originally manufactured by Aspect Medical Systems, Norwood, MA, USA). The EEG data from 3 of these patients had to be excluded from this analysis due to either technical problems during data acquisition (*n* = 1) or incomplete preoperative cognitive data (*n* = 2), leaving 35 patients with both BIS EEG and preoperative cognitive data for analysis. The data output port on the A-2000 monitor was connected by cable to an ASUS laptop computer (Taipei, Taiwan), and the raw EEG data were recorded using software developed by Hagihira et al. (2001). This raw EEG was recorded with a pre-amplifier bandwidth of 0.25–100 Hz, sampling rate of 256 Hz, 16-bit depth, and a full-scale range of ±1.8 mV. The BIS Quatro electrode array records from locations approximately at frontal sites F7 (channel 1) and Fp1 (channel 2) in the International 10–20 system, with the reference electrode at Fpz and the ground electrode ~1 cm medial to channel 2. After recording, we focused on analyzing EEG data specifically from channel 1, since channel 1 is the primary BIS EEG channel, while channel 2 is used for artifact detection by the BIS monitor (personal communication with Medtronic staff, January 2017). Channel 1 data were high-pass filtered at 2 Hz (to remove artifactual drift). Following the method of Purdon et al. (2015a) for analyzing data from clinical forehead-based EEG monitors, for each patient we selected for analysis the first continuous 2-min segment of EEG, starting >10 min after anesthesia induction, that was free of artifacts and burst suppression and had stable EEG dynamics (i.e., not transitioning to burst suppression). These 2-min segments were identified an average of 21.1 min after anesthesia induction (SD = 14.8 min), but it is possible that some patients’ data segments began before surgical incision, which has been shown to affect alpha power (Kochs et al., 1994). The individual performing this data selection was blinded to the patient’s cognitive data.

**Figure 1:**
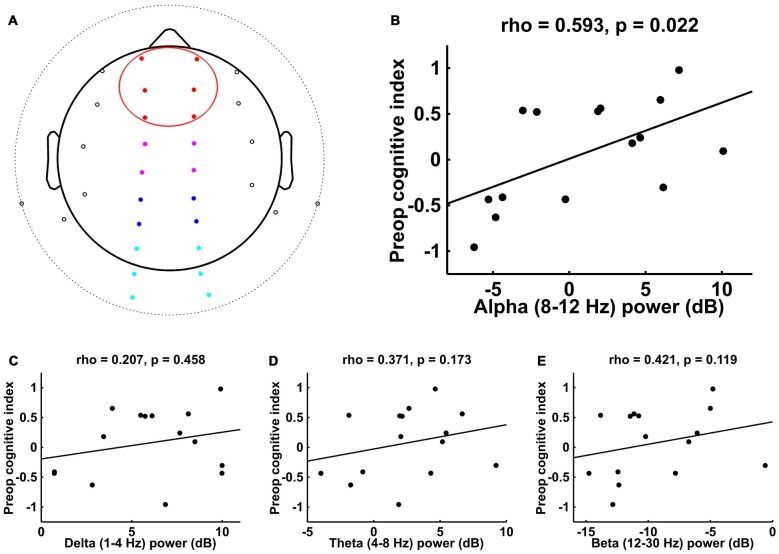
**Spearman correlations between preoperative cognitive index score and intraoperative frontal electroencephalogram (EEG) power in different frequency bands. (A)** Electrode locations for 32-channel EEG. Electrodes that are beyond the horizon of the head from this top view appear outside of the circular head schematic. Red denotes the frontal electrode region of interest (ROI; the data for which are plotted in **B–E**); other ROIs include central (magenta), parietal (blue), and occipital (cyan). **(B)** Alpha (8–12 Hz) showed a significant correlation with preoperative cognitive index score, while **(C–E)** delta (1–4 Hz), theta (4–8 Hz), and beta (12–30 Hz) did not.

For both the 32-channel and BIS data sets, we computed the power spectrum for each patient using the fast Fourier transform (FFT) method with a Hanning window taper and window length of *T* = 3 s with no overlap between epochs, implemented in the FieldTrip toolbox (Oostenveld et al., 2011). We then converted the average absolute power values to a decibel scale by taking 10*log_10_(*Power_f_*). For each patient, we focused on calculating the average power within the alpha band (8–12 Hz); in addition, we calculated the average power within the delta (1–4 Hz), theta (4–8 Hz), and beta (12–30 Hz) frequency bands. We divided the 32-channel data into four electrode regions of interest (ROIs)—frontal, central, parietal, and occipital (see Figure 1A)—and calculated the average power within each ROI.

### Cognitive Assessment

Preoperative neurocognitive assessment was performed using our well-established neurocognitive test battery (Newman et al., 2001; Mathew et al., 2013; Browndyke et al., 2016), which includes the following tests: the Randt Short Story Memory Test, the Modified Visual Reproduction Test from the Wechsler Memory Scale, the Digit Span Test from the revised version of the Wechsler Adult Intelligence Scale (WAIS-R), the Digit Symbol Test from the WAIS-R, and the Trail Making Test, Part B. The scores from these tests were then combined using factor analysis with orthogonal rotation (a linear transformation of the data) to produce uncorrelated cognitive domain factor scores, which were derived in a previous study of 394 similar non-cardiac surgical patients (McDonagh et al., 2010). This approach identified four cognitive domains: (1) verbal memory; (2) abstraction and visuospatial orientation (executive function); (3) visual memory; and (4) attention and concentration, which together accounted for 84% of the total test variability in our previous study (McDonagh et al., 2010). These four cognitive domain scores were then averaged together to provide the overall cognitive index scores reported here. The overall cognitive index score mean was zero, with a positive score representing better than average overall cognitive testing performance, and vice versa.

### Statistical Analysis

To test our primary hypothesis, we calculated the Spearman’s rank-order correlation between the average intraoperative frontal alpha-power values and the cognitive index scores derived from preoperative cognitive testing for patients with 32-channel EEG data. For patients with single-channel frontal EEG data recorded from the BIS sensor, we analyzed the relationship between alpha power and preoperative cognitive function, also using Spearman’s rank-order correlation. Although there is evidence that inhaled anesthetics such as isoflurane lead to different EEG dynamics—particularly in the alpha and theta bands—than intravenous ones such as propofol (Purdon et al., 2015b), we grouped together patients who received either isoflurane or propofol for anesthetic maintenance due to the relatively small number of patients who had usable EEG data and cognitive data (*n* = 15 for the 32-channel group, *n* = 35 for the BIS group). Future studies with larger, *a priori* determined sample sizes will likely be necessary to determine whether the magnitude of the correlation between intraoperative frontal alpha power and preoperative cognitive index scores differs between patients who receive isoflurane vs. propofol for anesthetic maintenance.

We derived the average age-adjusted end-tidal isoflurane MAC fraction and the average BIS index values for each patient based on the minute-to-minute values recorded from procedure start to procedure end. As these values are subject to signal contamination and other sources of noise, we first filtered the minute-to-minute values by calculating the median of a moving window of 5-min epochs during the time from procedure start to procedure end. We then transformed the filtered median end-tidal isoflurane concentration values in each 5-min window into the age-adjusted end tidal isoflurane MAC fraction, using 1.17 as the MAC value for isoflurane with a 6% decline in MAC per decade after age 30 (Mapleson, 1996). The case average age-adjusted end-tidal isoflurane MAC fraction and average BIS index value for each patient was defined as the mean of these 5-min median values; see Tables 1, 2 for this data.

For comparison with our focus on frontal alpha, we also performed *post hoc* analyses in which we calculated Spearman correlations between preoperative cognitive index scores and frontal EEG power in the other frequency bands (i.e., delta, theta and beta). Furthermore, to gain insight into the topographic distribution of the alpha effects, we also performed a *post hoc* exploratory analysis of the correlations between preoperative cognitive index scores and alpha power in the other predefined ROIs (i.e., central, parietal, and occipital). Lastly, we created topographic heatmaps of the alpha-cognition correlation values across all 32 channels to examine the alpha effects across the entire scalp. Analyses were performed with MATLAB (version R2013b, The MathWorks, Inc., Natick, MA, USA), and statistical significance was set at *α* = 0.05.

## Results

The EEG electrode locations for the 15 patients who underwent 32-channel recordings are shown in Figure 1A, with the frontal ROI of focus here highlighted in red. The analysis of the EEG activity from these patients revealed a significant correlation between intraoperative frontal alpha power and preoperative cognitive index score (Figure 1B; *r*_S_ = 0.593, *p* = 0.022). To assess whether this correlation was specific to the alpha band or whether it might simply reflect a more general decrease in broadband EEG power in patients with lower preoperative cognitive function, we examined whether frontal EEG power in other frequency bands would similarly correlate with preoperative cognitive function. These analyses indicated that intraoperative frontal EEG power in the delta, theta, and beta frequency bands did not show a significant correlation with preoperative cognitive index score (Figures 1C–E, respectively).

To assess the topographic distribution of the alpha effects, particularly in regard to the hallmark anesthesia-induced anteriorization pattern, we measured the correlations between preoperative cognitive index score and intraoperative alpha power in the other partitioned ROIs (central, parietal and occipital—see “Materials and Methods” Section and Figure 1A). These analyses showed that, in addition to the frontal ROI, alpha power in both the central and parietal ROIs showed a significant correlation with preoperative cognitive function across patients (central: *r*_S_ = 0.661, *p* = 0.009; parietal: *r*_S_ = 0.575, *p* = 0.027), whereas activity in the occipital ROI did not show such a correlation (*r*_S_ = 0.271, *p* = 0.327).

**Figure 2:**
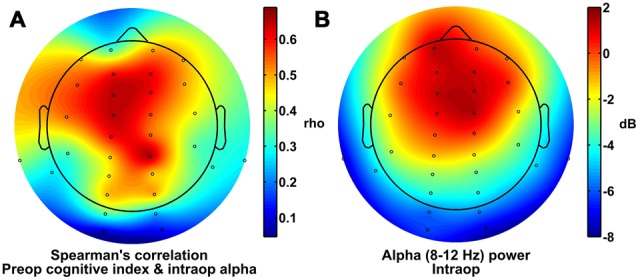
**Scalp topography of alpha power and correlation with cognitive index scores. (A)** Spearman correlation coefficients for comparisons between preoperative cognitive index score and intraoperative alpha power at each electrode site. **(B)** Alpha power under general anesthesia.

In addition, for illustrative purposes and to gain insight into the topographic distribution of the alpha effects, we also calculated the Spearman correlation coefficients (i.e., rho values) between preoperative cognitive index scores and the average intraoperative alpha power at each electrode across patients, and plotted these correlation coefficients as a topographic heatmap (Figure 2A). These values showed a broad maximum over frontal, central, and parietal sites, a scalp topography that was qualitatively similar to the fronto-central topography of alpha power measured intraoperatively (Figure 2B). Further, both of these topographies (Figures 2A,B) appear qualitatively different than the topography of alpha power measured preoperatively with eyes closed, which has the hallmark parieto-occipital maximum for the awake, eyes-closed state (Berger, 1929; Barry et al., 2007).

**Figure 3:**
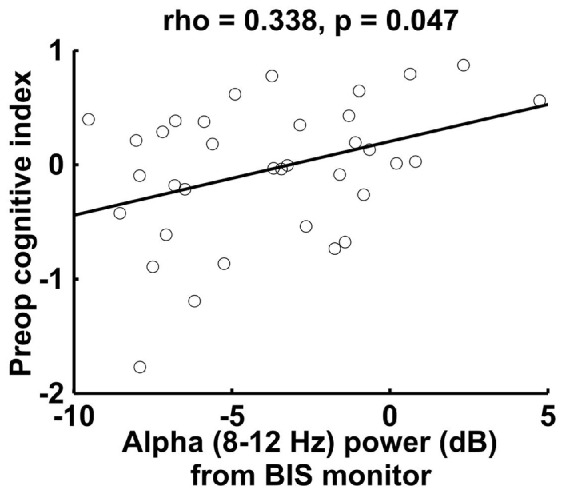
**Spearman correlation between preoperative cognitive index score and intraoperative alpha power measured by the bispectral index (BIS) monitor in a separate sample of 35 patients**. The BIS alpha power shown here is lower than the 32-channel data alpha power (Figure 1B), most likely due to the proximity of the reference electrode for the BIS montage (also on the forehead) (For the 32-channel data, we referenced to the algebraic average of the mastoid electrodes).

To further corroborate the correlation between intraoperative frontal alpha power and preoperative cognitive function, we analyzed data from a separate sample of 35 patients who underwent intraoperative single-channel frontal EEG data collection from the BIS. In this patient group, we also found that frontal alpha power correlated significantly with preoperative cognitive index scores (Figure 3; *r*_S_ = 0.338, *p* = 0.047). These data thus provide additional support for and corroboration of our findings for the frontal ROI of the 32-channel data in an independent patient sample.

## Discussion

Here we demonstrate in 50 older adults anesthetized with propofol or isoflurane that intraoperative frontal alpha power correlates with preoperative cognitive function. In the 15 patients who underwent 32-channel EEG recordings, such a correlation between frontal alpha-band activity and preoperative cognitive function was not observed in other EEG frequency bands (i.e., delta, theta, or beta). Further, the alpha-power correlation that was seen in the frontal ROI extended back to the central/parietal ROIs, but was not observed at occipital electrode sites.

The topography of scalp regions at which we found strong correlations between intraoperative alpha power and preoperative cognitive status (Figure 2A) is similar to the topography of alpha power under general anesthesia (Figure 2B). This increased frontal alpha power under general anesthesia in normal individuals reflects a large-scale shift in alpha power distribution—and in the thalamocortical dynamics that likely underlie it—from the awake state to the anesthetized state. Awake individuals typically show higher alpha power at parietal and occipital sites and lower alpha power over frontal ones, particularly when the eyes are closed, whereas anesthetized patients show the opposite pattern (i.e., alpha anteriorization; Tinker et al., 1977; John et al., 2001; Purdon et al., 2013). Thus, one interpretation of these results is that the brains of patients with worse preoperative cognitive function are less able to undergo the changes during anesthesia that result in the typical alpha anteriorization pattern. Our results suggest that such a failure to undergo anteriorization, or to show significant frontal alpha power, under general anesthesia is an intraoperative, electrophysiological marker of poorer preoperative cognitive status. Since poor preoperative cognitive status is itself a risk factor for POCD (Bekker et al., 2010; Silbert et al., 2015; reviewed in Berger et al., 2015) and may be a risk factor for postoperative delirium (Greene et al., 2009; but also see Scott et al., 2015), this finding raises the possibility that a failure of the brain to manifest strong frontal alpha power under general anesthesia may also be a predictor of POCD and postoperative delirium. If future studies provide evidence for such associations between lower intraoperative frontal alpha power and postoperative delirium and/or POCD, then these results would suggest that lower intraoperative frontal alpha power (and deficient anteriorization) could be used as a real-time and relatively inexpensive intraoperative electrophysiological marker to identify patients at potential increased risk of postoperative delirium and/or POCD, and could be used to target these at-risk patients for potential therapeutic intervention and/or for increased postoperative follow-up care and monitoring.

Aside from the question of whether lower intraoperative frontal alpha power may serve as a predictor of postoperative delirium and/or POCD, another fundamental question is why some older patients display lower intraoperative frontal alpha power. One potential explanation for this finding could be frontal cortical atrophy in some older patients (McGinnis et al., 2011; Fjell et al., 2014), which could result in an increased physical distance between the cortical sources of the EEG signals and the electrodes at the scalp surface (Hämäläinen et al., 1993). This hypothetical increased physical distance, however, would cause an attenuation of the entire broadband EEG signal measured at the scalp, and not just alpha specifically. Previous research has found an age-related decrease in power across all frequencies under general anesthesia, but with a relatively larger decrease for alpha power specifically (Purdon et al., 2015a). If cortical atrophy is the primary contributor to both decreased EEG power and lower cognitive function, one would expect that power in other frequency bands, such as delta (1–4 Hz), would also correlate with preoperative cognitive status. However, we did not observe such a correlation, suggesting that the mechanism for decreased alpha power in older adults with lower preoperative cognitive status is not simply increased cortical atrophy in these patients.

If frontal cortical atrophy does not account for the correlation between intraoperative alpha power and preoperative cognitive function, then what does? We believe there are four main possibilities that might explain this correlation. Since the frontally distributed alpha power under general anesthesia is thought to be generated by a thalamocortical network (Ching et al., 2010; Purdon et al., 2013; Vijayan et al., 2013) similar to awake occipital alpha (Lopes da Silva et al., 1973, 1980; Steriade et al., 1990; Hughes and Crunelli, 2005; Hughes et al., 2011), lower alpha power could occur secondary to: (1) a thalamic problem; (2) a cortical problem; (3) some combination of both; or (4) an impaired thalamocortical dynamic.

Previous research including computational modeling suggests that the thalamus plays a necessary role in a reciprocal thalamocortical activity loop that generates alpha oscillations (Lopes da Silva et al., 1980; Steriade et al., 1990; Ching et al., 2010; Hughes et al., 2011; Purdon et al., 2013; Vijayan et al., 2013); thus, deficits in thalamic structure or function might cause decreased frontal alpha power under general anesthesia. The thalamus is responsible for nearly all input to the cortex—including sensory information (except olfaction), motor inputs from structures like the cerebellum and basal ganglia, inputs from limbic structures such as the hippocampus, and widespread modulatory inputs from structures involved in regulating arousal and the sleep-wake cycle—and is thus integral to proper neurocognitive function. While full thalamic lesions usually cause a near-total loss of consciousness (Castaigne et al., 1981; Blumenfeld, 2010; Berger and García, 2016), more subtle insults can lead to deficits in working memory, attention, and perception that can be detected by cognitive testing (Blumenfeld, 2010).

Alternatively, the idea that a cortical issue (e.g., altered or reduced cortical activity) might underlie lower intraoperative alpha power in patients with poor preoperative cognitive function would be consistent with the idea that aside from the thalamocortical loop proposed to generate alpha oscillations, these alpha oscillations can also be generated directly by intra-cortical circuits involving deep-layer pyramidal neurons (Silva et al., 1991; Castro-Alamancos, 2000; Sun and Dan, 2009; Buffalo et al., 2011; van Kerkoerle et al., 2014), potentially interacting with low-threshold spiking interneurons (Vierling-Claassen et al., 2010). Thus, a potential deficit in the structure or function of these frontal cortical circuits themselves could result in lower intraoperative frontal alpha power.

Finally, it is also possible that some combination of thalamic and cortical deficits, and/or their functional or anatomic connectivity, might underlie both lower preoperative cognitive function and lower intraoperative frontal alpha power. In fact, some age-dependent neurodegenerative processes (such as the deposition of amyloid beta plaque and/or tau tangles in AD) may impair synaptic transmission within both the thalamus and cortex, and the process of biological aging itself may impair the function of synapses in both the thalamus and cortex (Henley and Wilkinson, 2013). Further, surgery and anesthesia cause neuroinflammation (Yeager et al., 1999; Buvanendran et al., 2006; Bromander et al., 2012; Hirsch et al., 2016), which may impair synaptic transmission and neural activity (Avramescu et al., 2016), and it is possible that there is a larger neuroinflammatory response in the brains of patients with lower preoperative cognitive function. Taken together, any combination of these mechanisms could conceivably alter connectivity/synaptic transmission in the thalamus and cortex and between these structures, which could play a role in causing the lower intraoperative frontal alpha power we have observed in patients with lower preoperative cognitive function.

Much future research clearly will be required to better understand the neurobiological underpinnings of why some patients have lower intraoperative frontal alpha power, and to understand why lower alpha power correlates with poor preoperative cognitive status. Additional research will also be needed to determine whether lower intraoperative frontal alpha power is associated with specific postoperative complications (such as POCD, delirium, or decreased quality of life) and/or changes in postoperative mortality rates. A limitation of the current study is that, due to our relatively small sample sizes, we grouped together patients who received either isoflurane or propofol for anesthetic maintenance, although there is evidence that these different anesthetics lead to different EEG dynamics (Purdon et al., 2015b). Future studies with larger, *a priori* determined sample sizes will likely be necessary to determine whether either the magnitude of the correlation between intraoperative frontal alpha power and preoperative cognitive index scores, or the lack of such a significant correlation for the other frequency bands, differs between patients who receive isoflurane vs. propofol for anesthetic maintenance. In sum, the present data suggest that intraoperative anteriorization and frontal alpha power correlate with preoperative cognitive function in older adults, and demonstrate a compelling need for future prospective studies to better understand the underlying mechanisms and associated sequelae of deficient intraoperative anteriorization and lower frontal alpha power in older adults.

## Author Contributions

MB and MGW conceived, designed, and oversaw the study. CMG, MB, and MGW organized and wrote the manuscript; All authors approved the final manuscript. JPM and JNB designed the cognitive test battery, and MC analyzed the cognitive data. CMG, KCR, JEG, EM and MB performed the 32-channel and BIS EEG recordings. EM and MB consented the study patients. CMG, KCR, JEG and FMS analyzed the 32-channel and BIS EEG data under the guidance of MB and MGW.

## Funding

This work was supported by National Institutes of Health (NIH) grants T32 #GM08600 (in part to MB), R03-AG050918 (to MB), a Jahnigen Award from the American Geriatrics Society and the Foundation for Anesthesia Education and Research (to MB), an International Anesthesia Research Society Mentored Research Award (to MB), Duke Anesthesiology departmental funds, and additional research funds from MGW.

## Conflict of Interest Statement

The authors declare that the research was conducted in the absence of any commercial or financial relationships that could be construed as a potential conflict of interest. The reviewer MK and handling Editor declared their shared affiliation, and the handling Editor states that the process nevertheless met the standards of a fair and objective review.
